# Supplementary material for: A novel setup for simultaneous two-photon functional imaging and precise spectral and spatial visual stimulation in Drosophila
Source: Sci Rep. 2020 Sep 24;10:15681. doi: 10.1038/s41598-020-72673-5 (PMC7515906; doi:10.1038/s41598-020-72673-5)
Supplement: Supplementary file 1 — Supplementary information [file 41598_2020_72673_MOESM1_ESM.pdf]

A novel setup for simultaneous two-photon functional imaging and precise spectral and spatial visual stimulation in *Drosophila*

Authors: R. C. Feord[1] & T. J. Wardill[1,2*]

Affiliations:

[1]Physiology, Development & Neuroscience, University of Cambridge, CB2 3EG, UK.

[2]Ecology, Evolution and Behavior, University of Minnesota, St Paul, MN, 55108, USA.

*Correspondence to: twardill@umn.edu

SUPPLEMENTARY INFORMATION

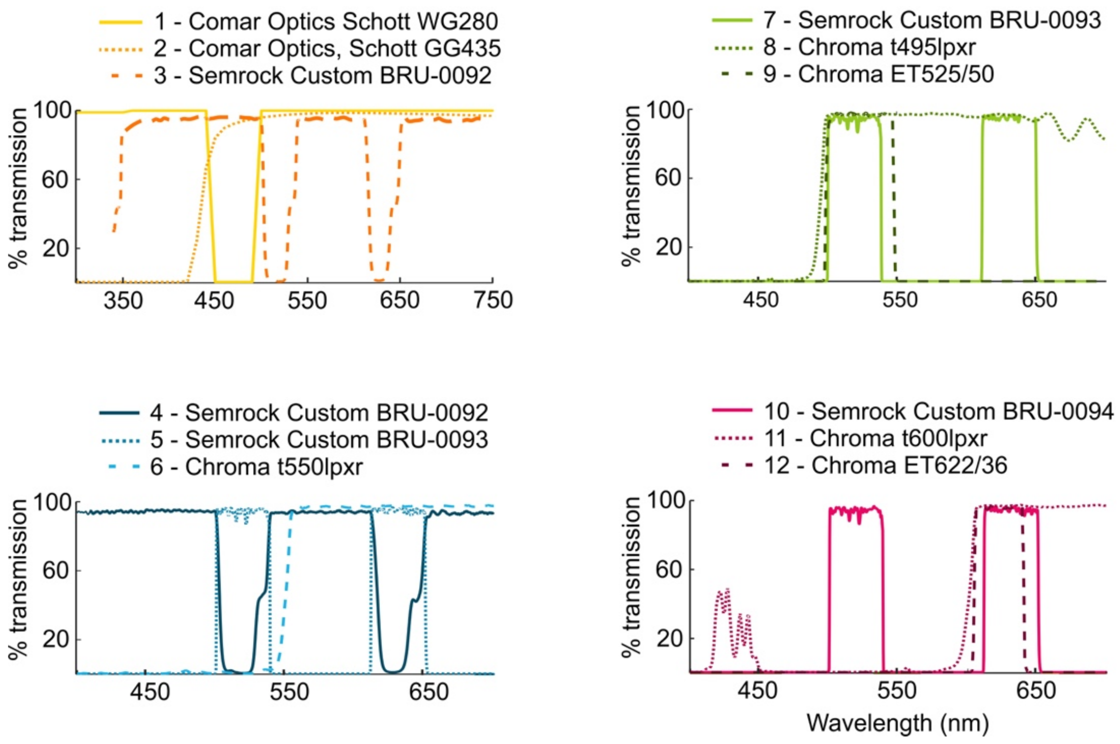

Figure S1. Filter spectra for the monochromator and the microscope.
*Filter spectra for the modified optical pathways in the monochromator and the microscope depicted in **Figure 1B**.*

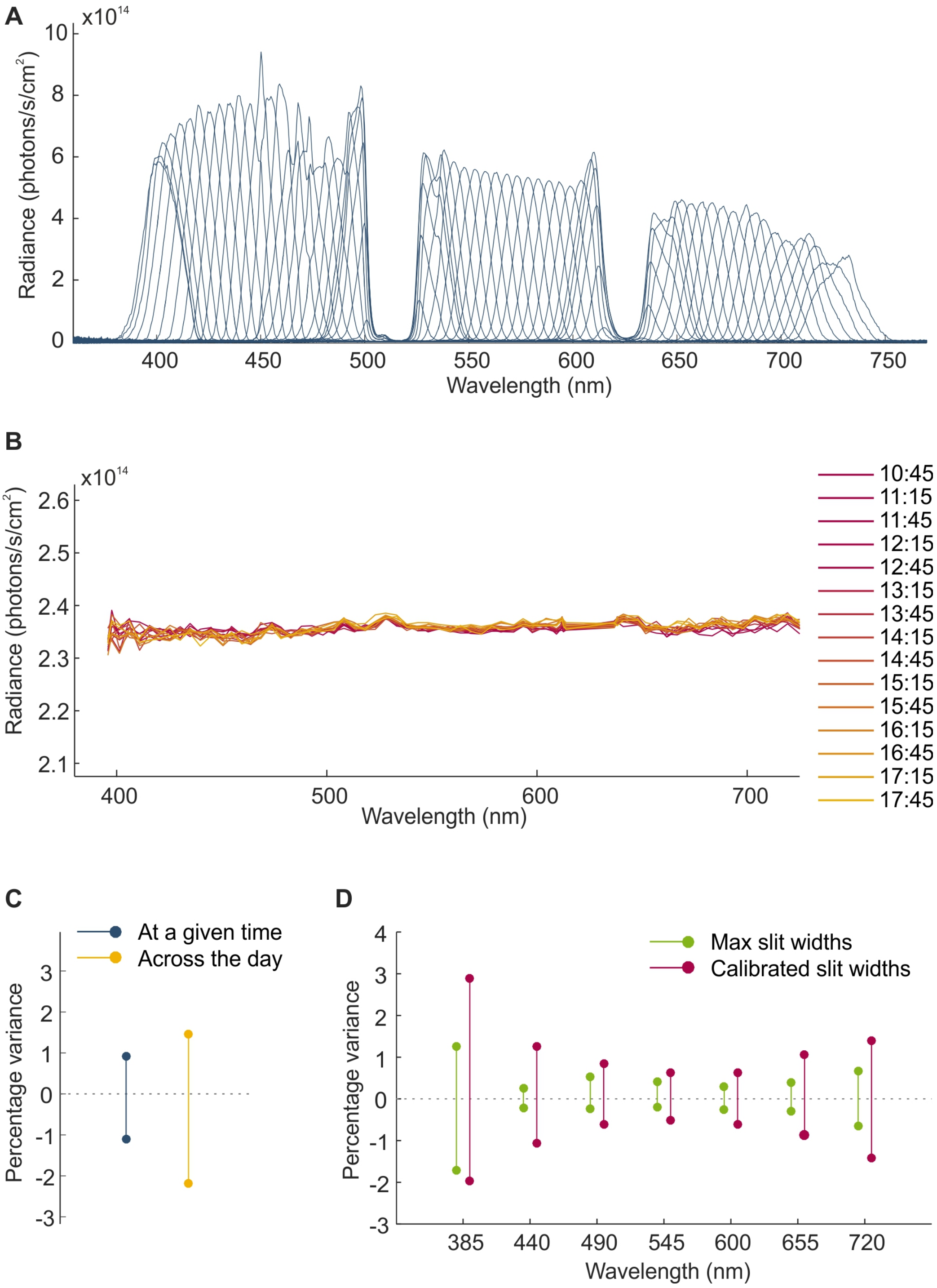

Figure S2. Detailed optical specifications of the monochromator-projector setup.
*(**A**) Radiance curves for each centre wavelength (385 to 720 nm in approximate 5 nm increments) for monochromator input and exit slits calibrated to produce equal brightness corresponding to a set reference value (here the radiance of the dimmest band of light, 385 nm centre wavelength, for a 30 nm slit width). (**B**) Calibrated radiance values determined from the area under the curve for each radiance spectra, measured at half hour intervals across the experimental day. (**C**) Brightness fluctuation of calibrated light across the spectrum reported as the maximum and minimum percentage variance from the mean at any given time (see B), and over the course of the entire day. (**D**) Power fluctuations for individual centre wavelengths at maximum slit width and calibrated slit widths over the course of 5 minutes. Percentage change corresponds to maximum and minimum change from the mean of measurements taken every second over the course of five minutes.*

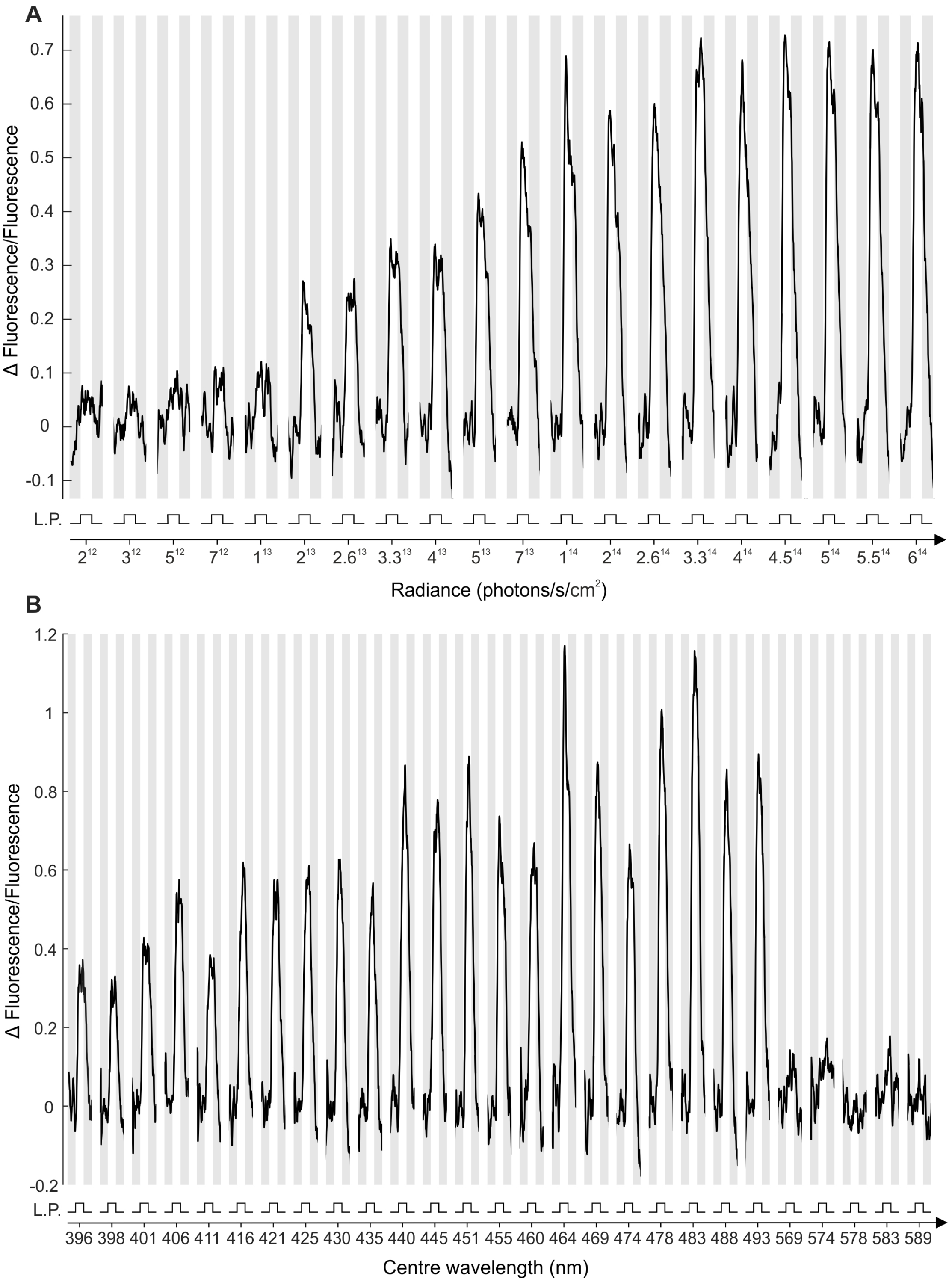

Figure S3. Intensity-response and spectral sweep stimuli: example responses.
*(**A**) Example fluorescence responses of an ROI to the intensity-response stimulus protocol consisting of light pulses (L.P.) of increasing intensity. Traces for a given intensity represent the mean of the response from all three stimulus repeats. (**B**) Example fluorescence responses of an ROI to the spectral sweep stimulus protocol consisting of light pulses of varying centre wavelengths. Traces for a given intensity represent the mean of the response from all three stimulus repeats. Response traces are not represented beyond 590 nm for the sake of clarity as no discernible change in fluorescence is detected beyond this point. Note that pulses were presented randomly but have been reordered here in ascending wavelength.*

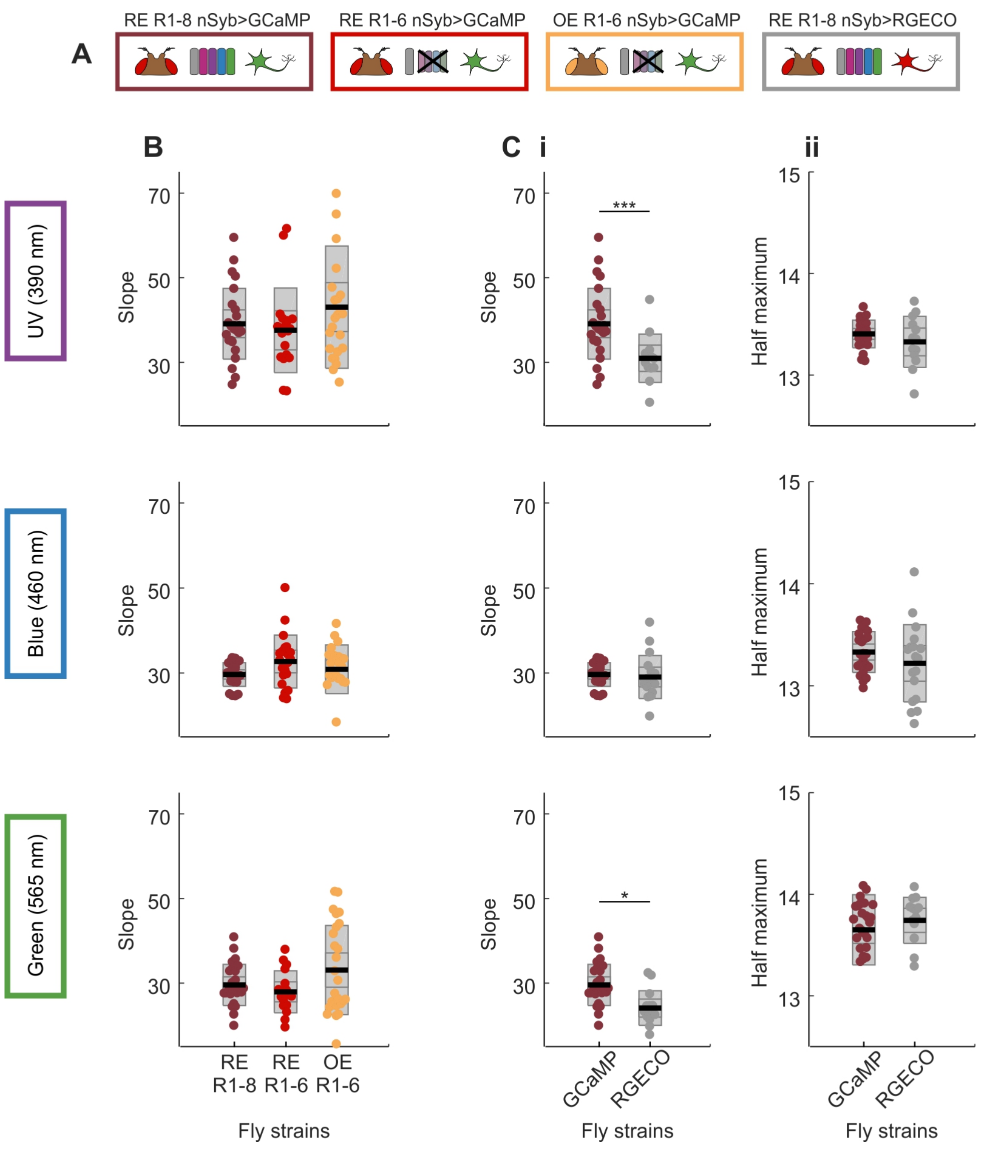

Figure S4. Intensity-response relationship coefficient comparison across fly strains.

*(**A**) Experiments were performed in four different fly strains expressing pan-neuronal calcium activity indicators– red eye, wild type photoreceptors and GCaMP6f (RE WT(R1-R8), nSyb>GCaMP6f, bordeaux); red eye, Rh1 only and GCaMP6f (RE R1-R6, nSyb>GCaMP6f, red); orange eye, Rh1 only and GCaMP6f (OE R1-R6, nSyb>GCaMP6f, orange) and red eye, wildtype photoreceptors and RGECO (RE WT (R1-R8), nSyb>RGECO, grey). (**B**) Slope values extracted from intensity-response relationship curves for three different bands of monochromatic light (UV, blue and green; centre wavelengths 390, 460 and 565 nm respectively) for GCaMP-expressing fly strains. (**C**) Slope (**i**) and half maximum radiance (photons/s/cm^2)(**ii**) values extracted as in (B) comparing responses in red eye/wild type photoreceptors flies expressing either GCaMP6f or RGECO. Individual data points correspond to the average of ROI responses across a given layer structure (see methods) for a given fly preparation. The curves in (A) are a mean of the fitted curves of individual data points. Black line = mean, inner grey box = SEM, outer grey box = SD. Significant differences are noted with star values, with the P values from top to bottom: ***P = 0.0005 and *P = 0.0415 (one-way ANOVA).*

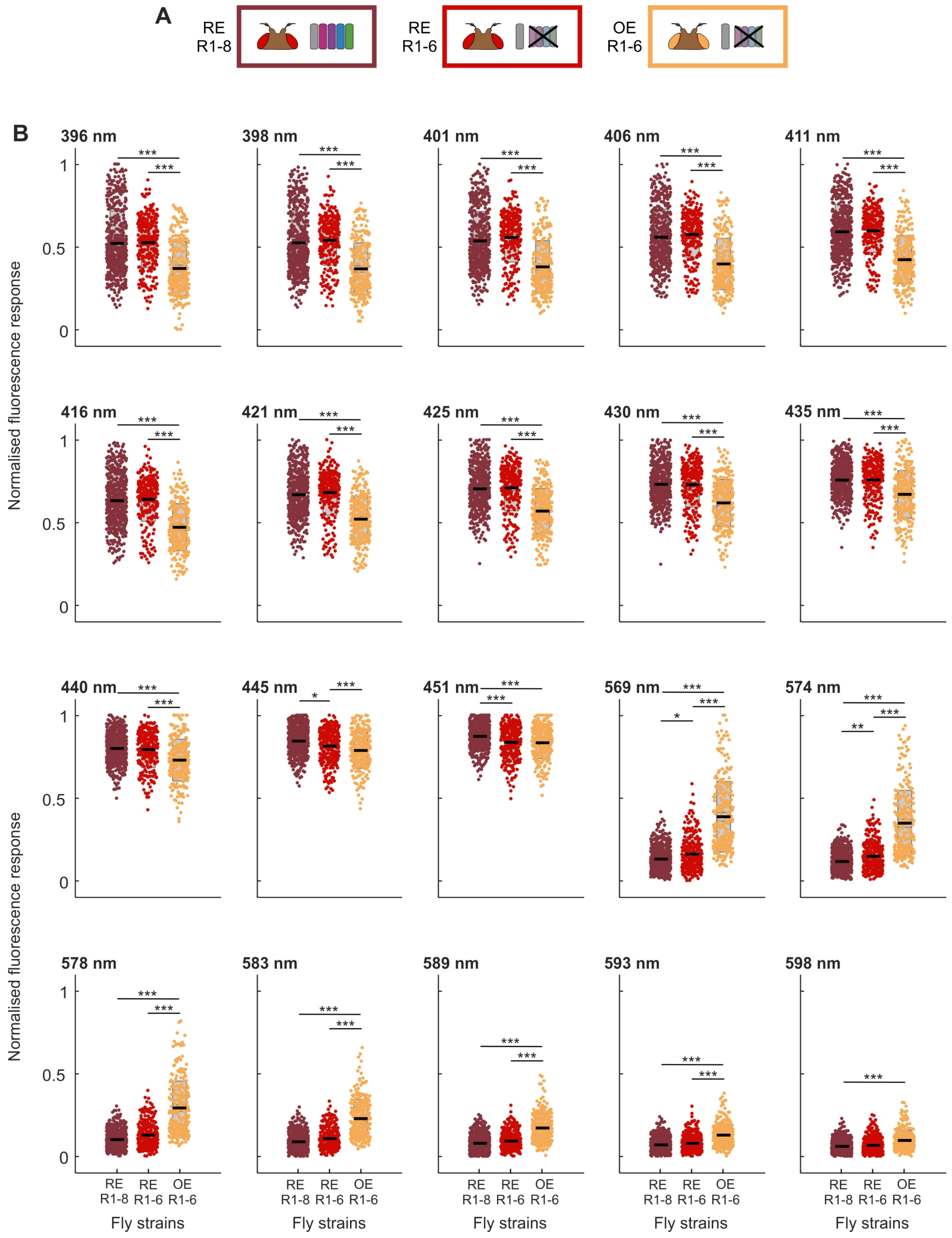

Figure S5. Spectral response profiling – breakdown for individual wavelengths

*(**A**) Fly strains expressing pan-neuronal GCaMP6f– red eye/wild type photoreceptors (RE WT(R1-R8), nSyb>GCaMP6f, bordeaux); red eye/Rh1 only(RE R1-R6, nSyb>GCaMP6f, red); orange eye/Rh1 only(OE R1-R6, nSyb>GCaMP6f, orange). (**B**) Responses to the individual wavelengths of the spectral sweep in Figure 3D that exhibit significant differences. Black line = mean, inner grey box = SEM, outer grey box = SD. Significant differences are noted with star values, with one-way ANOVA P values reported in table S1.*

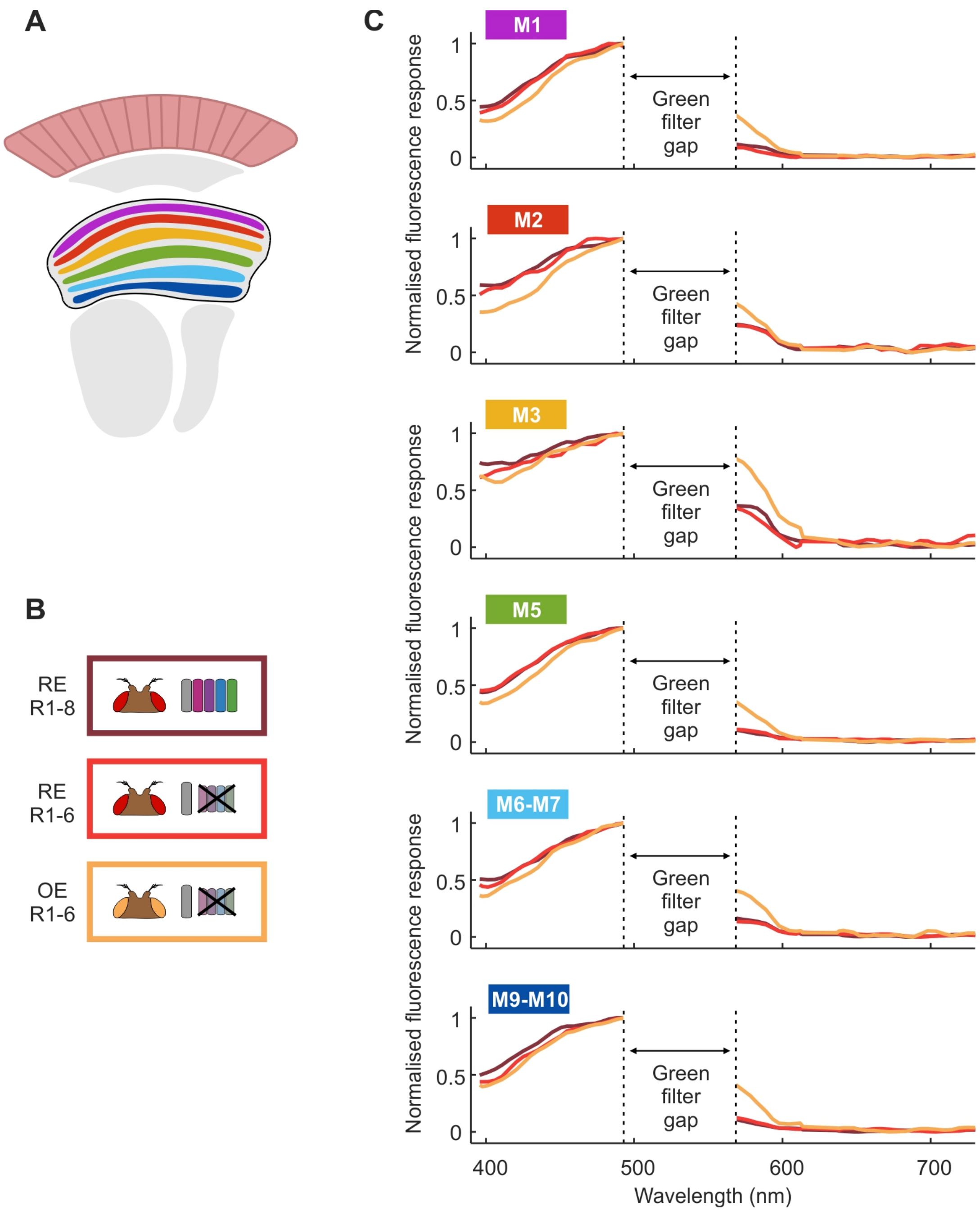

Figure S6. Spectral response profiles of layer groupings in the medulla

*(**A**) Schematic of layer structures in the medulla as distinguished from pan-neuronal GCaMP labelling (see Figure 4). (**B**) Fly strains expressing pan-neuronal GCaMP6f– red eye/wild type photoreceptors (RE WT(R1-R8), nSyb>GCaMP6f, bordeaux); red eye/Rh1 only (RE R1-R6, nSyb>GCaMP6f, red); orange eye/Rh1 only (OE R1-R6, nSyb>GCaMP6f, orange). (**C**) Mean spectral response profiles for layers M1, M2, M3, M5, M6-M7 and M9-M10.*

Table S1. 1-way ANOVA p-values from Figure 3D and Figure S5B

	RE-WT/RE-Rh1	RE-WT/OE-Rh1	RE-Rh1/OE-Rh1
**396**	1	3.2094e-05	3.2094e-05
**398**	1	3.2094e-05	3.2094e-05
**401**	0.8996	3.2094e-05	3.2094e-05
**406**	0.9999	3.2094e-05	3.2094e-05
**411**	1	3.2094e-05	3.2094e-05
**416**	1	3.2094e-05	3.2094e-05
**421**	1	3.2094e-05	3.2094e-05
**425**	1	3.2094e-05	3.2094e-05
**430**	1	3.2094e-05	3.2094e-05
**435**	1	3.2094e-05	3.2094e-05
**440**	1	3.2094e-05	3.2094e-05
**445**	0.0294	0.6676	3.2094e-05
**451**	1.9732e-04	4.3811e-05	1
**569**	0.0283	3.2094e-05	3.2094e-05
**574**	0.0098	3.2094e-05	3.2094e-05
**578**	0.1550	3.2094e-05	3.2094e-05
**583**	0.9891	3.2094e-05	3.2094e-05
**589**	1	3.2094e-05	3.2094e-05
**593**	1	3.2094e-05	3.2309e-05
**598**	1	2.5220e-04	0.2610

Table S2. 1-way ANOVA p-values from Figure 4C

		RE-WT/RE-Rh1	RE-WT/OE-Rh1	RE-Rh1/OE-Rh1
**M1**	UV	1	1	1
	Blue	1	0.0039	0.6421
	Green	1	7.2533e-06	7.2533e-06
**M2**	UV	7.2533e-06	1	7.2533e-06
	Blue	7.2533e-06	0.7172	7.2533e-06
	Green	7.2533e-06	7.2533e-06	7.2533e-06
**M3**	UV	0.9962	1	0.9960
	Blue	0.4919	1	0.9999
	Green	0.9957	0.3603	1
**M5**	UV	1	0.1753	0.9403
	Blue	1	1	1
	Green	1	7.2534e-06	7.2534e-06
**M6-M7**	UV	1	0.0335	0.9671
	Blue	1	0.2098	0.9167
	Green	1	1	1
**M9-M10**	UV	1	0.0022	0.8434
	Blue	0.9999	1	0.9981
	Green	1	7.2534e-06	7.2534e-06
